# Current status of insecticide resistance in malaria vectors in the Asian countries: a systematic review

**DOI:** 10.12688/f1000research.46883.1

**Published:** 2021-03-10

**Authors:** Dewi Susanna, Dian Pratiwi

**Affiliations:** 1 Department of Environmental Health, Faculty of Public Health, Universitas Indonesia, Depok, Jawa Barat, 16424, Indonesia; 2 Alumni of Faculty of Public Health, Universitas Indonesia, Depok, Jawa Barat, 16424, Indonesia

**Keywords:** Anopheles; Malaria Elimination; Vector Control Program; Insecticide Resistance; Asian Countries

## Abstract

**Background** **:** The application of insecticides for malaria vector control has remained a global problem, due to the current trend of increased resistance against these chemicals. This study aims to determine the insecticide-resistant status in Asia and how to implement the necessary interventions. Moreover, the implications of resistance in malaria vector control in this region were studied.

**Methods:** This systematic review was conducted using a predefined protocol based on PRISMA-retrieved articles from four science databases, namely ProQuest, Science Direct, EBSCO, and PubMed in the last ten years (2009 to 2019). The searching process utilized four main combinations of the following keywords: malaria, vector control, insecticide, and Asia. In ProQuest, malaria control, as well as an insecticide, were used as keywords. The following criteria were included in the filter, namely full text, the source of each article, scholarly journal, Asia, and publication date as in the last ten years.

**Results: **There were 1408 articles retrieved during the initial search (ProQuest=722, Science Direct=267, EBSCO=50, PubMed=285, and Scopus=84). During the screening, 27 articles were excluded because of duplication, 1361 based on title and abstract incompatibility with the inclusion criteria, and 20 due to content differences. In the final screening process, 15 articles were chosen to be analyzed. From the 15 articles, it is known that there was dichlorodiphenyltrichloroethane (DDT) and pyrethroids resistance in several anopheles species with a mortality rate of less than 80%.

**Conclusion** **s** **:** The report on the pyrethroid resistance was complicated, since this insecticide was considered effective in malaria vector control. Therefore, several strategies were required, including the management plans in selecting insecticides, using a rotation system during interventions in the field, regular monitoring, and integrating vector control based on physics, chemistry, and biology. All of these need to be supported by cross-sector policies and cooperation in achieving the 2030 malaria-free target.

## Introduction

Malaria is one of the most common vector-borne diseases widespread in the tropics and subtropics ^1^. According to the World Malaria Report (2019), an estimated 219 million cases were recorded in 2017, compared to the 217 million in the previous year ^2^. The global estimate of deaths caused by malaria reached 435,000 cases, which was the same number in 2016 ^3^. The use of insecticides is the basis for the effective control of vectors, and this process has played an essential role in the management and elimination of malaria ^4^.

The prevention of malaria mainly depends on one class of insecticides, namely pyrethroids; however, the increase in resistance to it reduces this treatment's efficacy ^5^. The progressive reduction of malaria's burden through substantial improvements of insecticide-based vector control in recent years is reversible by the emergence of widespread resistance to this chemical ^6^. Insecticide resistance is widespread and is now reported in almost two-thirds of the countries with ongoing malaria transmission. This resistance affects all major vector species and groups of insecticides ^7^.

Vector control is an essential aspect of a program organized to manage the disease transmitted by this type of organism. The use of insecticides for this process is an effective strategy; however, it is also related to the development of resistance in targeted vectors and is one reason for the failure of disease control in many countries ^8^. Since 2000, malaria cases have halved due to the management and vector control interventions, estimated to have saved 660 million people ^9^. The global commitment to eliminate malaria by 2030 requires immediate efforts that include the establishment of infrastructure for monitoring regular insecticide resistance, the development of combined and effective control products ^10^.

This review aimed to determine the status of insecticide resistance in Asia and how to implement interventions. It is also expected that this sets an example for other countries in the vector control program and provides guidance for insecticides and malaria risk reduction.

## Methods

### Search strategy

This study retrieved articles from four science databases, namely ProQuest, Science Direct, EBSCO, and PubMed, from December 2009 to December 2019. A systematic review was conducted using a predefined protocol based on the preferred reporting items for systematic reviews and meta-analyses (PRISMA) ^11, 12^. The searching process utilized four main combinations of the following keywords: “malaria”, “vector control”, “insecticide”, and “Asia”. In order to reduce the risk of bias from the articles obtained, the researchers conducted disbursements in all databases using the same keywords and on the same day.

In ProQuest, “malaria” and “vector control”, as well as “insecticide”, were used as keywords. The full text, the source of an article, scholarly journal, Asia, and date of publication as in the last ten years were included in the filter. The search strategy and filter used in Science Direct were the same as that above except "Asia". In EBSCO, a similar keyword was also used. The limiters were the same as the filter in the Proquest, but also included "abstract available". In the Pubmed, the terms used were as follows, ("malaria"[MeSH Terms] OR "malaria"[All Fields]) AND ("vector"[MeSH Terms] OR "vector"[All Fields]) AND ("control"[MeSH Terms] OR "control"[All Fields]) AND ("insecticide"[MeSH Terms] OR "insecticide"[All Fields]) AND ("loattrfulltext"[sb] AND "2009/12/02"[PDat] : "2019/12/02"[Pdat])s.

### Inclusion and exclusion criteria

Original articles (academic or research papers) in Asia, written in English and published in the last ten years were included. Study designs such as prospective study, review, cross-sectional, cohort, and case control were included. Articles about biochemical, resistance to dieldrin (RDL) mutation, knowledge and attitudes, and spatial modeling were excluded because that can cause different results. Articles about malaria but including nothing about insecticides were excluded. The implications of insecticide resistance in related countries were investigated. Studies that were not relevant to this study were excluded.

### Study selection

The articles' eligibility was determined from each title, abstract, and full text by two reviewers (DP and DS). DP and DS also independently screened the articles for inclusion and extracted data on general information. To solve any disagreements and problems during the study, regular meetings were held by the researchers to discuss issues.

### Data extraction and analysis

The search strategy and inclusion and exclusion criteria were validated and implemented. The initial database was then created from the electronic search. All citations were first filtered by title and abstract, and duplicates omitted. The full texts of eligible papers were then obtained independently for further filtering. After resolving the differences in data extraction or interpretation through consensual discussions based on the inclusion and exclusion criteria mentioned above, the final papers were selected.

The data from the chosen eligible studies were the authors, study period, publishing year, the country where it was conducted, settings, location characteristics, bioassay methods, the sample of Anopheles mosquito, and the quartiles in SCImagoJR. SCImagoJR is a publicly available portal that includes the journals and country scientific indicators developed from the information contained in the Scopus database. The findings were arranged according to the objective and result obtained in related implications of malaria vector control resistance. Throughout the entire selection process, the use of insecticides in the bioassay method, the associated mortality rate of the *Anopheles* mosquito, and its implementation in the specific areas were reported to illustrate the practice's pattern and extent.

All variables for which we extracted data ware Anopheles species, vector habitat, bioassay method, insecticides, mortality rate, insecticide resistance strategies/intervention. The differences in methods could bias the results; to reduce this bias, we selected articles with a similar method. For articles about insecticide resistance, we only looked at articles using bioassay with the world health organization (WHO) standard ^13^. The WHO bioassay is carried out with paper impregnated from four main classes of insecticides in common use, with different concentrations according to the WHO test procedure ^14^.

## Results

**Figure 1.**
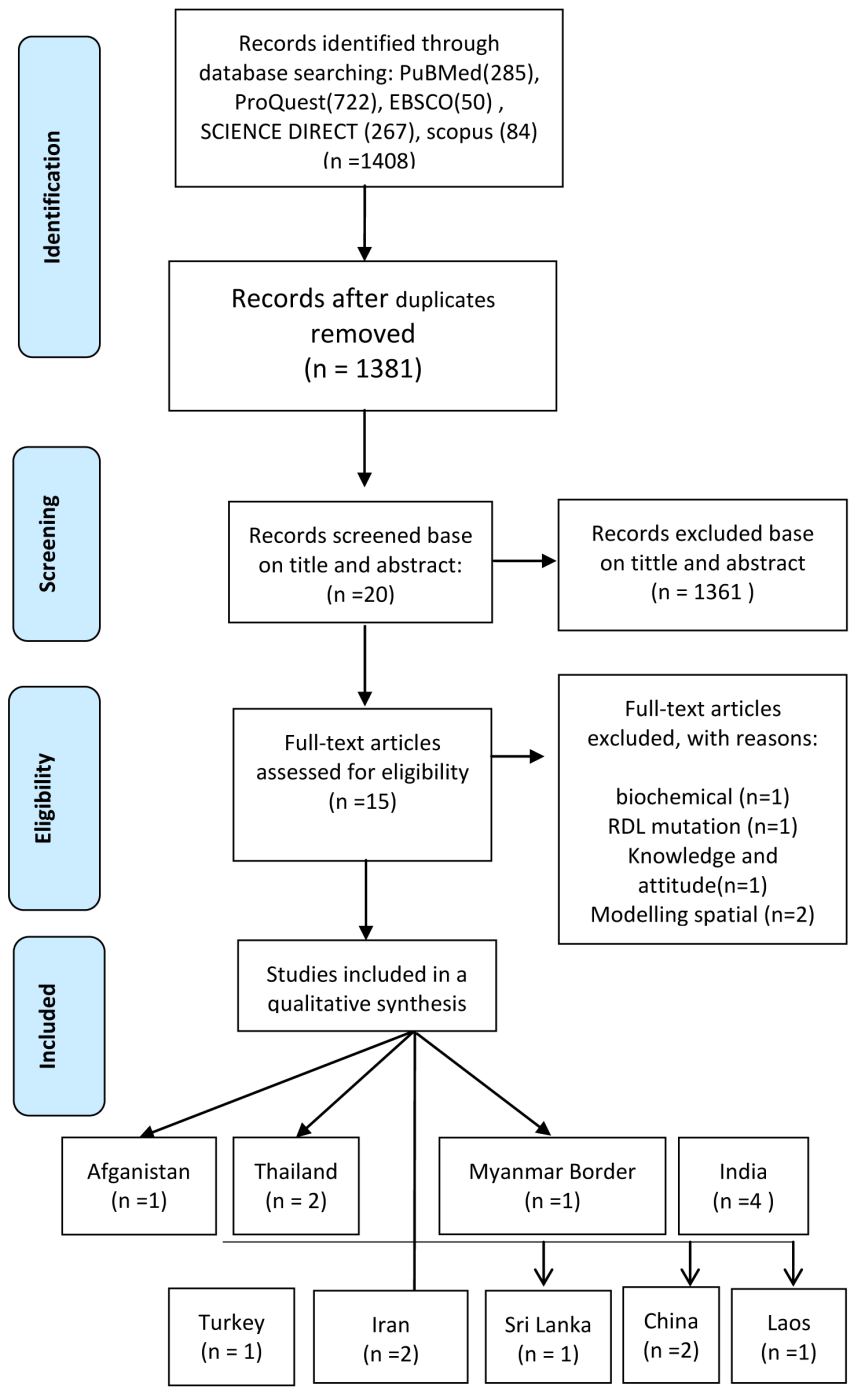
PRISMA flow diagram of systematic review inclusion and exclusion process.

The identification process for review is outlined in Figure 1 ^15^. There were 1,408 articles retrieved during the initial searching (ProQuest=722, Science Direct=267, EBSCO=50, Pubmed=285 and Scopus=84). Through screening, 27 articles were excluded because of duplication, 1,361 based on title and abstract incompatibility and 20 due to inconsistency with the inclusion criteria; 15 were chosen to be analyzed.

The 15 eligible articles originated from eight Asian countries published from 2012 to 2019 journals are shown in Table 1.

**Table 1.** Article characteristics.

Authors	Title	Publish	Country	Study periods	Publisher	Reference
Ahmad *et al.*	Status of insecticide resistance in high-risk malaria provinces in Afghanistan.	2016	Afghanistan	August to October 2014	Malaria Journal	24
Mishra *et al.*	Insecticide resistance status of *Anopheles culicifacies* in Madhya Pradesh, central India	2012	India	August to September 2009	Journal of Vector- Borne Diseases	8
Dhiman *et al.*	Insecticide resistance and human blood meal preference for *Anopheles annularis* in Asom-Meghalaya border area, northeast India	2014	India	June–August 2011	Journal of Vector- Borne Diseases	21
Sahu *et al.*	Triple insecticide resistance of *Anopheles culicifacies*: A practical impediment for malaria control in Odisha State, India.	2015	India	April to June 2014	Indian Journal of Medical Research	19
Chand *et al.*	Insecticide resistance status of *An. culicifacies* in Gadchiroli (Maharashtra) India	2017	India	August 2016 and February 2017	Pathogens and global health	25
Chareonviriyaphap *et al.*	Review of insecticide resistance and behavioral avoidance of human diseases by vectors in Thailand	2013	Thailand	2000–2010	Parasites & Vectors	26
Chaumeau *et al.*	Insecticide resistance of malaria vectors along the Thailand-Myanmar border	2017	Thailand	August and November 2014, July 2015	Parasites & Vectors	20
Sumarnrote *et al.*	Insecticide resistance status of Anopheles mosquitoes in Ubon Ratchathani province, Northeastern Thailand	2017	Thailand	September 2013–September 2015	Malaria Journal	17
Gorouhi *et al.*	Biochemical Basis of Cyfluthrin and DDT Resistance in *Anopheles stephensi* (Diptera: Culicidae) in Malarious Area of Iran	2018	Iran	April–June 2015	Journal of Arthropod-Borne Diseases	22
Vatandoost *et al.*	Indication of pyrethroid resistance in the main malaria vector, *Anopheles stephensi* from Iran	2012	Iran	Spring 2011	Asian Pacific Journal of Tropical Medicine	27
Marcombe *et al*.	Insecticide resistance status of malaria vectors in Lao PDR	2017	Lao	The rainy (June to October) and dry (January to May) seasons of 2014 and 2015	PloS One	28
Qin *et al*.	Insecticide resistance of *Anopheles sinensis* and An. vagus in Hainan Island, a malaria-endemic area in China	2014	China	July–August 2012	Parasites & Vectors	29
Dai *et al.*	Development of insecticide resistance of malaria vector *Anopheles sinensis* in Shandong Province In China	2015	China	2003–2012	Malaria Journal	30
Surendran *et al.*	Variations in susceptibility to common insecticides and resistance mechanisms among morphologically identified species of the malaria vector *Anopheles subpictus* in Sri Lanka	2012	Sri Lanka	July 2008–June 2010	Parasites & Vectors	31
S.İ. Yavaşoglu, *et al.*	Current insecticide resistance status of *Anopheles sacharovi* and *A. superpictus* in former malaria-endemic areas of Turkey	2019	Turkey	April 2014 and September 2015	ActaTropica	32

There were 23 species of Anopheles from these studies ( Table 2). In several countries, the main vectors included *An. stephensi* (Iran), *An. superpictus* (Afghanistan), *An. culicifacies* (India), *An. minimus* and *An. maculatus* (Lao and Thailand), *An. sinensis* (China), *An. subpictus* (Sri Lanka), and *An. sacharovi* (Turkey). From Table 2, the malaria vector was divided into three habitats, based on distribution, namely in rice fields, forests and on the coast, and flowing rivers. The differences in the main vector of each country depended on environmental/ecological conditions, living habitat, as well as the feeding and resting behavior of each *Anopheles*.

**Table 2.** Sample and location characteristics.

Study location	Country	Anopheles species	Sample adult female mosquitos (n)	Vector habitat	Reference
Nangarhar, Laghman, Kunar, Ghazni, and Badakhshan	Afghanistan	*An. stephensi*, *An. superpictus*, *An. culicifacies*	2049	Ricefield, river stream, ponds, and water puddle	24
Madhya Pradesh	India	*An. culicifacies*	NA	Forest	8
Asom-Meghalaya border area, northeast India	India	*An. annularis*	200	Forest, ponds irrigation	21
Rayagada, Nowrangpur, Kalahandi, Malkangiri and Koraput	India	*An. culicifacies*	1740	Forest	19
Gadchiroli district	India	*An. culicifacies*	NA	Forest	25
Chiang Mai-Chiang Dao, Mae Hongsom, Phrae	Thailand	*An. minimus* *An. annularis*	NA	Paddy fields and rivulet	26
Thailand-Myanmar Border	Thailand	*An. annularis*, *An. minimus*, *An. hyrcanus*, *An. barbirostris*, *An. vagus*, *An. maculatus*, *An. jamessi*, *An. scanloni*, *An. kochi*, *An. tesselatus*	5896	Agriculture	20
Khong Chiam, Sirindhorn, Buntharik, and Nachaluay	Thailand	*An. hyrcanus*, *An. barbirostris*, *An. maculatus*, *An. nivipes*, *An. philipinensis*, *An. vagus* *An. dirus* *An. karwari*	2088	Forest and ricefield	17
Chabahar Seaport, southeast corner of Iran	Iran	*An. stephensi*	317	Seaport	22
Sistan and Baluchistan	Iran	*An.stephensi*	733	Coastal	27
Phongsaly, Bokeo, LuangPrabang, Vientiane Pro, Borlikhamxay, Khammouane, Savannakhet, Saravane, Sekong, Attapeu.	Lao	*An. minimus*, *An. hyrcanus*, *An. vagus* *An. maculatus* *An. nivipes* *An. philipinesnis* *An. umbrosus* *An. kochi* *An. tesselatus* *An. aconitus*	3977	Forest, village, agriculture	28
Hainan Island	China	*An. sinensis*, *An. vagus*	1468	Mountainous and ricefield	29
Shandong Province	China	*An. sinensis*	4370	Irrigated ricefield, aquatic habitat, and small ponds	30
Batticaloa, Puttalam, Trincomalee and Ampara	Sri Lanka	*An. subpictus* *An. sundaicus*	256	Coastal and inland	31
Southeastern Anatolia and the Mediterranean	Turkey	*An. superpictus*, *An. sacharovi*	1230	Agricultural, ponds, stream and swamps	32

The entire female *Anopheles* collected was morphologically identified for their species or complexes using stereomicroscopes and morphological keys ^16^. The mosquitoes were separated into groups of species, complexes, or bioassay, then kept alive by giving them a sugar solution ^17^.

*Anopheles* mosquitoes were morphologically identified at the adult stage using the Glick identification key ^18^. The susceptibility tests were carried out following the WHO guidelines for monitoring resistance in malaria vectors. From 15 papers reviewed, the impregnated with insecticides of DDT (4%), malathion (5%), bendiocarb (0.1%), propoxur (0,1%), deltamethrin (0.05%) and l-cyhalothrin (0.05%), cyfluthrin 0.15%, permethrin 0.75%, and etofenprox 0.5% was prepared by adopting the WHO standard method ^14^ ( Table 3).

**Table 3.** WHO bioassay method.

Country	WHO bioassay with insecticides								
	Organochlorine	Organophosphate	Carbamate		Pyrethroid				
	DDT (%)	malathion (%)	Bendiocarb (%)	Propoxur (%)	Permethrin (%)	Deltamethrin (%)	Lambda- cyhalothrin (%)	Cyfluthrin (%)	Etofenprox (%)
Afghanistan	4.0	5.0	0.1	NA	0.75	0.05	NA	NA	NA
India	4.0	5.0	NA	NA	NA	0.05	NA	NA	NA
India	4.0	NA	NA	NA	NA	0.05	NA	NA	NA
India	4.0	5.0	NA	NA	NA	0.05	NA	NA	NA
India	4.0	NA	NA	NA	0.75	0.05	0.05	0.15	NA
Thailand	4.0	NA	NA	NA	NA	NA	NA	NA	NA
Thailand	4.0	NA	NA	NA	0.75	0.05	NA	NA	NA
Thailand	4.0	NA	NA	NA	0.75	0.05	NA	NA	NA
Iran	4.0	NA	NA	NA	0.75	0.05	0.05	0.15	0.5
Iran	4.0	NA	NA	NA	0.75	0.05	0.05	0.15	0.5
Lao	4.0	NA	NA	NA	0.75	0.05	NA	NA	NA
China	4.0	5.0	NA	NA	NA	0.05	NA	NA	NA
China	4.0	5.0	NA	NA	NA	0.05	NA	0.15	NA
Sri Lanka	4.0	5.0	NA	NA	NA	0.05	0.05	NA	NA
Turkey	4.0	5.0	NA	0,1	0,75	0,05	NA	NA	0.5

WHO=world health organization. DDT=dichlorodiphenyltrichloroethane. NA= not available.

The insecticide bioassay was then carried out using a recommended standard WHO kit ^13^. The mortality rate was recorded 24 hours after exposure, while the average death was calculated for each insecticide and according to the WHO criteria ^19^. Bioassay results were summarized in three resistance classes according to the WHO criteria ^12^: (1) susceptible when death was 98% or higher, (2) resistance-possibility was tolerable when mortality was between 97% and 80%, and (3) resistant when death case was lower than 80% ^18^.

The highest mortality rate (MR) ≥ 98% of etofenprox application was on *An. stephensi* in Iran. While permethrin application was on *An. superpictus* (Afghanistan), *An. nivipes* (Thailand), *An. philipinensis* (Lao and Thailand), and *An. tesselatus (*Lao). Then, bendiocarb and malathion were on *An. culicifacies* (India and Afghanistan). Also, deltamethrin was on *An. anularis* (India), *An. barbirostris*, *An. dirus*, *An. karwari* (Thailand), *An. vagus* (Thailand and China), *An. maculatus* (Lao and Thailand), and *An. umbrosus* (Lao). *An minimus* (Thailand Myanmar Border), Meanwhile, permethrin and deltamethrin were on *An. aconitus*, and *An. kochi* (Lao). Lastly, lambda cyalothrin and deltamethrin were on *An. sundaicus* (Sri Lanka), with MR ≤ 97% indicating resistance-possibility based on the WHO classification.

Table 4 shows the level of anopheles resistance to organochlorine (dichlorodiphenyltrichloroethane; DDT), organophosphate (malathion), carbamate (bendiocarb and propoxur), and pyrethroid (permethrin, deltamethrin, lambda cyalothrin, cyfluthrin, and etofenprox). Almost all the species of this mosquito studied were possibly resistant to DDT. Furthermore, this similar issue has been reported in *An. stepensi*, *An. superpictus*, *An. culicifacies*, *An. vagus*, *An. sinensi*, *An. subpictus*, and *An. sachrovi* to malathion. Also, it was found in *An. superpictus* and *An. sachrovi* to propoxur as well as in *An. umbrosus* to permethrin. The same was in *An. sinensis*, *An. superpictus*, *An. sundaicus*, *An. minimus*, *An. maculatus* and *An. jamessi* to deltamethrin. Resistance was also reported in *An. stephensi*, *An. culcifacies*, *An. vagus*, and *An. barbirostris* to permethrin and deltamethrin, and in *An. stephensi* to etofenprox. However, direct resistance was found in *An. hyrcanus* to Permethrin and deltamethrin, as well as in *An. culicifacies* to lambda cyalothrin. Also, this was found in *An. stephensi*, *An. sinensis* and *An. culicifacies* to cyfluthrin.

**Table 4.** Mortality rate of insecticide resistance bioassay in *Anopheles*.

No	Anopheles species	Percentage of mortality (Susceptibility Status)									Reference
		Organochlorine	Organophosphate	Carbamate		Pyrethroid					
		DDT (%)	Malathion (%)	Bendiocarb (%)	Propoxur (%)	Permethrin (%)	Deltamethrin (%)	Lambda- cyhalothrin (%)	Cyfluthrin (%)	Etofenprox (%)	
1	An. stephensi ^*^	31-60 **(R)**	47-97 **(R)**	87-100 (PR)	NA	87-91 (PR)	66-78 **(R)**				24
		45 **(R)**	NA	NA	NA	92.3 (PR)	96(PR)	88.4 (PR)	55 **(R)**	91 (PR)	27
		62 **(R)**	NA	NA	NA	NA	96 (PR)	89 (PR)	82 (PR)	100 (S)	22
2	An. superpictus ^*^	50-86.7 (R-PR)	61.7-88.3 (R-PR)		68.3-91.7 (R-PR)	NA	NA	NA	NA	NA	32
		100 (S)	100 (S)	92 (PR)	NA	100 (S)	85 (PR)	NA	NA	NA	24
3	An. culicifacies ^*^	6.6-26.6 **(R)**	65.4-100 (R-S)	NA	NA	NA	71.6-94.1 (R-PR)	NA	NA	NA	8
		11.4-15.3 **(R)**	60.4-76.2 **(R)**	NA	NA	NA	72.6-84 (R-PR)	NA	NA	NA	19
		37.1 **(R)**	74 **(R)**	NA	NA	91.3 (PR)	83.8 (PR)	59.9 **(R)**	70.2 **(R)**	NA	25
		81 (PR)	95 (PR)	100 (S)		89 (PR)	64 **(R)**				24
4	*An. annularis*	11.9-28.3 **(R)**	NA	NA	NA	NA	97.7-98.1(PR-S)	NA	NA	NA	21
		NA	NA	NA	NA	NA	NA	NA	NA	NA	26
							100 (S)				20
5	An. minimus ^*^	NA	NA	NA	NA	NA	NA	NA	NA	NA	26
		NA	NA	NA	NA	NA	92 (PR)	NA	NA	NA	20
		98-100 (S)	NA	NA	NA	100 (S)	100 (S)	NA	NA	NA	28
6	An. hyrcanus ^*^	57 **(R)**	NA	NA	NA	48 **(R)**	33 **(R)**	NA	NA	NA	20
		72-83 (R-PR)	NA	NA	NA	65-87 (R-PR)	45-85 (R-PR)	NA	NA	NA	17
		90 (PR)	NA	NA	NA	NA	NA	NA	NA	NA	28
7	An. barbirostris ^*^	69 **(R)**	NA	NA	NA	NA	97-100 (PR-S)	NA	NA	NA	17
		74 **(R)**	NA	NA	NA	84 (PR)	72 **(R)**	NA	NA	NA	20
8	An. vagus ^*^	34-61 **(R)**	NA	NA	NA	89-95 (PR)	79-95 (R-PR)	NA	NA	NA	28
		67.1-88.8 (R-PR)	77.3-88.9 (R-PR)	NA	NA	NA	NA	NA	NA	NA	29
		97 (PR)	NA	NA	NA	95 (PR)	75 **(R)**	NA	NA	NA	20
		NA	NA	NA	NA	NA	97.9-100 (S)	NA	NA	NA	29
		NA	NA	NA	NA	NA	100 (S)	NA	NA	NA	17
9	An. maculatus ^*^	86-100 (PR-S)	NA	NA	NA	NA	NA	NA	NA	NA	28
		NA	NA	NA	NA	97 (PR)	85 (PR)	NA	NA	NA	20
		NA	NA	NA	NA	NA	100 (PR)	NA	NA	NA	28
		NA	NA	NA	NA	NA	100 (PR)	NA	NA	NA	17
10	*An. jamessi*	NA	NA	NA	NA	NA	87 (PR)	NA	NA	NA	20
11	*An. nivipes*	0-100 (R-S)	NA	NA	NA	90-100 (PR)	100 (S)	NA	NA	NA	28
		NA	NA	NA	NA	100 (S)	NA	NA	NA	NA	17
12	*An. philippinenses*	33-100 (R-S)	NA	NA	NA	100 (S)	NA	NA	NA	NA	28
		100 (S)	NA	NA	NA	100 (S)	100 (S)	NA	NA	NA	17
13	*An. umbrosus*	63 **(R)**	NA	NA	NA	86 (PR)	100 (S)	NA	NA	NA	28
14	An. sinensis ^*^	30.4 **(R)**	86.6 (PR)	NA	NA	NA	35.8 **(R)**	NA	32.4 **(R)**	NA	30
		72.7-78.4 **(R)**	NA	NA	NA	NA	85.8-91(PR)	NA	NA	NA	29
15	An. subpictus ^*^	16-35 **(R)**	49-69 **(R)**	NA	NA	NA	82-96 (PR)	72-97 (R-PR)	NA	NA	31
16	An. sacharovi ^*^	55-78.3 **(R)**	58.3-90 (R-PR)	NA	68.3-90 (R-PR)	NA	NA	NA	NA	NA	32
17	*An.scanloni*	84 (PR)	NA	NA	NA	NA	NA	NA	NA	NA	20
18	*An.kochi*	82-100 (PR-S)	NA	NA	NA	100 (S)	100 (S)	NA	NA	NA	28
		NA	NA	NA	NA	NA	98 (S)	NA	NA	NA	20
19	*An.tessellatus*	14 **(R)**	NA	NA	NA	100 (S)	NA	NA	NA	NA	28
		NA	NA	NA	NA	NA	98 (S)	NA	NA	NA	20
20	*An.sundaicus*	38-47 **(R)**	93-98 (PR-S)	NA	NA	NA	97-100 (S)	100 (S)	NA	NA	31
21	*An.aconitus*	100 (S)	NA	NA	NA	100 (S)	100 (S)	NA	NA	NA	28
22	*An.dirus*	NA	NA	NA	NA		100 (S)	NA	NA	NA	17
23	*An.karwari*	NA	NA	NA	NA		100 (S)	NA	NA	NA	17

*Main vector DDT=dichlorodiphenyltrichloroethane. NA=not available, S=Susceptible (90-97% mortality suggest), P=Possible Resistance, R=Resistance= < 90%.

Table 5 shows that insecticide resistance interventions in several Asian countries are through vector prevention by environmental, biological, and chemical management. Implementation is through insecticide rotation monitoring, mapping, and surveillance. Malaria eradication started from effective prevention, technical capability approaches, government and community support, funding sources, accurate data, and adequate implementation.

**Table 5.** Insecticide resistance strategies.

Country	Study location	Location characteristic	Insecticide resistance strategies	Reference
Afganistan	Nangarhar, Laghman, Kunar, Ghazni, and Badakhshan	Rice field, river stream, ponds, and water puddle	Establishing a management plan for insecticide resistance, and monitoring this situation in all malaria-endemic provinces.	24
India	Madhya Pradesh	Forest	Resistance management strategy by appropriate rotation of different insecticides, including carbamates and incorporating a synergist with synthetic pyrethroids for treating mosquito nets for the control of malaria vectors in these areas. Periodical monitoring of susceptibility/ resistance status of different insecticides.	8, 19, 21, 25
	Asom-Meghalaya border area, northeast India	Forest, ponds irrigation		
	Rayagada, Nowrangpur, Kalahandi, Malkangiri and Koraput	Cattle sheds, human dwelling		
	Gadchiroli district	Forest		
Thailand	Chiang Mai-Chiang Dao, Mae Hongsom, Phrae	Paddy fields and rivulet	Vector prevention strategies and monitoring insecticide resistance. Achieving universal coverage and proper use of LLIN for all people at risk of malaria. Alternative control tools (e.g., insecticide-treated clothes, spatial repellents, or treated hammocks) adapted to the situation of people's activities are more effective in reducing the malaria burden	17, 20, 26
	Thailand-Myanmar Border	Agriculture		
	Khong Chiam, Sirindhorn, Buntharik, and Nachaluay	Forest and rice field		
Iran	Chabahar Seaport, southeast corner of Iran	Seaport	Biological, chemical, and environmental management. Rotation of insecticide. Monitoring and mapping of insecticide resistance in the primary malaria vector for the implementation of any vector control. Evaluation of the mechanisms and implementation of proper insecticide resistance management strategies.	22, 27
	Sistan and Baluchistan	Coastal		
Lao	Phongsaly, Bokeo, LuangPrabang, Vientiane Pro, Borlikhamxay, Khammouane, Savannakhet, Saravane, Sekong, Attapeu.	Forest, village	Routine monitoring of the insecticide resistance levels and mechanisms to ensure effective malaria control. Use of insecticide with different modes of action, rotation, or combination in the same area.	28
China	Hainan Island	Mountainous and ricefield	Cost-effective integrated vector control programs that are beyond synthetic insecticides. The genetic basis of insecticide resistance to implementing more effective vector control strategies. Monitoring the efficacy of common insecticide and exploring the molecular basis of resistance.	29, 30
	Shandong Province	Irrigated ricefield, aquatic habitat, and small ponds		
Sri Lanka	Batticaloa, Puttalam, Trincomalee and Ampara	Coastal and inland	Monitoring genetically different vector populations and their sensitivity to varying insecticides. Developing simple molecular tools and techniques to differentiate morphologically similar anopheles species on the field.	31
Turkey	Southeastern Anatolia and the Mediterranean	Agricultural, ponds, stream, and swamps	Effective management of insecticide resistance and monitoring of the status at a regular interval to prevent delay to its development. Integrated vector control strategies including biological, chemical, and physical strategies implemented in a combination	32

## Discussion

### Study sites

Ecologically, the sites used were mountainous, harbor/seaport, mixed thicket/ lush and dense forests, humid climate, rivers, rice fields, and ponds that provide a suitable environment for vector mosquito breeding. Anopheles mosquitos' seasonal activity differs in various regions due to environmental conditions ^20^. Also, those collected were identified for species based on their morphological characteristics ^19, 21^.

### Type of insecticide

The application of chemical insecticides is one of the most critical interventions for malaria control, which included organochlorines (DDT, dieldrin, and BHC), organophosphates (pyrimytophos-methyl and malathion), carbamates (propoxur), and pyrethroids (lambda-cyhalothrin and deltamethrin). And were used in various forms of application, such as indoor residual spraying (IRS) and insecticide-treated mosquito nets (ITNS) for controlling adult mosquitos. In contrast, organophosphates for larviciding were used in malaria-prone areas ^22^. Currently, the pyrethroids were used in various Asia countries for ITNs and long-lasting insecticidal nets (LLINs). They were also considered the most effective because of their advantages, namely low mammalian toxicity, rapid knockdown activity, and high efficacy against a wide range of insect pests, especially mosquitos ^22^.

### Insecticide resistance level

Resistance to various insecticide classes was a common problem in different malaria vector species. This situation has been reported and is shown in Table 5, including widespread resistance to DDT and pyrethroids ^23^. The multiple resistance of reported malaria vector Included *An. stephensi*, *An. superpictus*, *An. culinary*, *An.annularis*, *An. minimus*, *An. hyrcanus*, *An. barbirostris*, *An. vagus*, *An. maculatus*, *An. jamessi*, *An nivipes*, *An. philippinensis*, *An. umbrosus*, and *An. sinensis* in Asia. Most of the new reports were towards pyrethroid compounds, the only insecticides used for LLINs ^22^. This became a challenge for malaria control and elimination, therefore, using the same insecticide for multiple successive IRS cycles is not recommended; preferably a rotation system with various types of these groups, including carbamates, should be used ^33^. The rotation should start with an insecticide that has the lowest resistance frequency. In high-coverage areas with LLINs, pyrethroids were good choices for IRS because this added to the selection pressure.

Furthermore, using an impregnated net synergistically with synthetic pyrethroid was a better choice for malaria vectors resistant to this class of chemical. The mixed nets, such as the aforementioned, have applications against resistible mosquitoes, mainly based on oxidative metabolism ^34^. Since monitoring of the resistance was a critical element for implementing insecticide-based vector control interventions, there was a need for periodic surveillance at least once a year or preferably every six months ^13^.

### Mortality rate of the *Anopheles*

The mortality test category was in the range between 98–100%, 80–97%, and <80%, and was categorized as susceptible, possibly resistant, and resistant population, respectively ^23^. All vectors had resistance to DDT with a value below 80%; however, the use of insecticide began to decline gradually over the last few decades and was removed entirely from malaria control in 2000. This decline was due to the perceived adverse effects on the environment and decreased public acceptance for spraying indoor residues ^35^.

Pyrethroids were the most commonly used insecticides for ITN and IRS, which target indoor transmission and mosquitos that bite in the room ^29^. The mortality rate was <80% for the pyrethroid group in *An. vagus*, *An. culinary*, *An. stephensi*, *An. hyrcanus*, *An. barbirostris*, *An. superpictus*, *An. sacharov*i, and *An. subpictus.* This proved that pyrethroid was less effective. Meanwhile, insecticide resistance was present in malaria vectors in Asia, and the genes spread rapidly throughout the world ^36^.

### Intervention

The use of insecticides to reduce vector populations has become the main strategy for malaria control. Presently, 12 of these interventions belonging to four chemical classes are recommended by the WHO Pesticide Evaluation Scheme (WHOPES) for IRS ^37^. Current strategies for controlling malaria vectors mainly include IRS with synthetic DDT/pyrethroids and durable LLINs ^14^. WHO recommends that these insecticides' susceptibility status needs to be monitored annually ^13^. However, the last two decades have seen the use of insecticides everywhere, especially pyrethroids, causing widespread resistance and compromising the effectiveness of vector control ^38^. Besides, when this situation is detected, the intensity, biochemical and molecular mechanisms should also be investigated. The accurate information about the underlying resistance mechanism and its intensity or frequency in the malaria vector turn to update the vector control program and ensure the timely management of insecticide resistance ^23^. Therefore, biochemical and molecular tests are recommended to understand the mechanism of pyrethroid resistance, and there have been several reports about this situation in the malaria vectors. However, several control strategies are used to fight resistance, such as rotation, mixture, using biological control, and integrated vector management ^27^.

### Limitations

This study had limitations, such as the dissimilar variables investigated, which produced an incomplete analysis. There were only 15 articles from eight countries that correlated with the inclusion criteria from the selected ten years of studies. The data on each country's mortality rate presented only the smallest value, therefore, making it difficult to explore the whole data that need to make the discussion complete. In some countries, the bioassay test did not use carbamate, even though it was effective for controlling certain types of Anopheles.

## Conclusion

The reports of pyrethroid resistance were quite focused on because it is considered effective in the malaria vector control. Several strategies are needed, including management plans in selecting insecticides, using a rotation system during the field interventions, regular monitoring, and integrating vector control based on physics, chemistry, and biology. All these need to be supported by cross-sector policies and cooperation to achieve the 2030 malaria-free target.

## Data availability

The data referenced by this article are under copyright with the following copyright statement: Copyright: ï¿½ 2021 Susanna D and Pratiwi D

Data associated with the article are available under the terms of the Creative Commons Attribution Licence, which permits unrestricted use, distribution, and reproduction in any medium, provided the original data is properly cited. 

### Underlying data

All data underlying the results are available as part of the article and no additional source data are required.

### Extended data

Figshare: PRISMA 2009 checklist for an article entitled Current status of insecticide resistance in malaria vectors in the Asian countries: systematic review. https://doi.org/10.6084/m9.figshare.13586078 ^15^. 

This project contains the following extended data:

- PRISMA flow diagram of systematic review inclusion and exclusion process

### Reporting guidelines

Figshare: PRISMA checklist for ‘Current status of insecticide resistance in malaria vectors in the Asian countries: systematic review’. https://doi.org/10.6084/m9.figshare.13582517 ^12^.

Data are available under the terms of the Creative Commons Attribution 4.0 International license (CC-BY 4.0).
