# Current status of insecticide resistance in malaria vectors in the Asian countries: a systematic review

**DOI:** 10.12688/f1000research.46883.2

**Published:** 2022-01-04

**Authors:** Dewi Susanna, Dian Pratiwi

**Affiliations:** 1 Department of Environmental Health, Faculty of Public Health, Universitas Indonesia, Depok, Jawa Barat, 16424, Indonesia; 2 Alumni of Master Program of Public Health, Universitas Indonesia, Depok, Jawa Barat, 16424, Indonesia

**Keywords:** Anopheles; Malaria Elimination; Vector Control Program; Insecticide Resistance; Asian Countries

## Abstract

**Background**: The application of insecticides for malaria vector control has led to a global problem, which is the current trend of increased resistance against these chemicals. This study aimed to review the insecticide resistance status was previously determined in Asia and how to implement the necessary interventions. Moreover, the implications of resistance in malaria vector control in this region were studied.

**Methods**: This systematic review was conducted using a predefined protocol based on PRISMA-retrieved articles from four science databases, namely ProQuest, Science Direct, EBSCO, and PubMed in the last ten years (2009 to 2019). The searching process utilized four main combinations of the following keywords: malaria, vector control, insecticide, and Asia. In ProQuest, malaria control, as well as an insecticide, were used as keywords. The following criteria were included in the filter, namely full text, the source of each article, scholarly journal, Asia, and publication date as in the last ten years.

**Results**: There were 1408 articles retrieved during the initial search (ProQuest=722, Science Direct=267, EBSCO=50, PubMed=285, and Scopus=84). During the screening, 27 articles were excluded because of duplication, 1361 based on title and abstract incompatibility with the inclusion criteria, and 20 due to content differences. In the final screening process, 15 articles were chosen to be analyzed. From the 15 articles, it is known that there was organochlorine (DDT), organophosphate (malathion), and pyrethroids resistance in several Anopheles species with a less than 80% mortality rate.

**Conclusion**: This review found multiple resistance in several Anopheles includes resistance to pyrethroid. The reports of pyrethroid resistance were quite challenging because it is considered effective in the malaria vector control. Several countries in Asia are implementing an insecticide resistance management (IRM) strategy against malaria vectors following the Global Plan for IRM.

## Introduction

Malaria is one of the most common vector-borne diseases widespread in the tropics and subtropics ^1^. According to the World Malaria Report (2020), an estimated that around 229 million cases of malaria in 2019 in 87 Malaria endemic countries, declined from 238 million in 2000 in 108 countries which were malaria endemic in 2000. Malaria death has been reduced in the period 2000–2019, from 736,000 in the year 2000 to 409,000 in 2019 ^2^. The global estimate of deaths caused by malaria reached 435,000 cases, which was the same number in 2016 ^3^. The use of insecticides is the basis for the effective control of vectors, and this process has played an essential role in the management and elimination of malaria ^4^.

The prevention of malaria depends on four classes of insecticides mailny one class of insecticides, namely pyrethroids; however, the increase in resistance to it reduces this treatment's efficacy and it is dangerous ^5^. The progressive reduction of malaria's burden through substantial improvements of insecticide-based vector control in recent years is partly reversible by the emergence of widespread resistance to this chemical ^6^. Insecticide resistance is widespread and is now reported in almost two-thirds of the countries with ongoing malaria transmission. This resistance affects all major vector species and groups of insecticides ^7^.

Vector control is an essential aspect of a program organized to manage the disease transmitted by *Anopheles* mosquitoes. The use of insecticides for this process is an effective strategy; however, it is also related to the development of resistance in targeted vectors and is one reason for the failure of disease control in many countries ^8^. Since 2000, malaria cases have halved due to the management and vector control interventions, estimated to have saved 660 million people ^9^. The global commitment to eliminate malaria by 2030 requires immediate efforts that include the establishment of infrastructure for regular monitoring insecticide resistance, the development of combined and effective control products ^10^.

This review aimed to determine the status of insecticide resistance in Asia and how to implement interventions. It is also expected that this sets an example for other countries in the vector control program and provides guidance for insecticides and malaria risk reduction.

## Methods

### Search strategy

This study retrieved articles from four science databases, namely ProQuest, Science Direct, EBSCO, and PubMed, from December 2009 to December 2019. A systematic review was conducted using a predefined protocol based on the preferred reporting items for systematic reviews and meta-analyses (PRISMA) ^11, 12^. The searching process utilized four main combinations of the following keywords: “malaria”, “vector control”, “insecticide”, and “Asia”. In order to reduce the risk of bias from the articles obtained, the researchers conducted disbursements in all databases using the same keywords and on the same day.

In ProQuest, “malaria” and “vector control”, as well as “insecticide”, were used as keywords. The full text, the source of an article, scholarly journal, Asia, and date of publication as in the last ten years were included in the filter. The search strategy and filter used in Science Direct were the same as that above except "Asia". In EBSCO, a similar keyword was also used. The limiters were the same as the filter in the ProQuest, but also included "abstract available". In the PubMed, the terms used were as follows, ("malaria"[MeSH Terms] OR "malaria"[All Fields]) AND ("vector"[MeSH Terms] OR "vector"[All Fields]) AND ("control"[MeSH Terms] OR "control"[All Fields]) AND ("insecticide"[MeSH Terms] OR "insecticide"[All Fields]) AND ("loattrfulltext"[sb] AND "2009/12/02"[PDat] : "2019/12/02"[Pdat])s.

### Inclusion and exclusion criteria

Original articles (academic or research papers) in Asia, written in English and published in the last ten years were included. Study designs such as prospective study, review, cross-sectional, cohort, and case-control were included. Articles about biochemical, resistance to dieldrin (RDL) mutation, knowledge and attitudes, and spatial modeling were excluded because that can cause different results. Articles about malaria but including nothing about insecticides were excluded. The implications of insecticide resistance in related countries were investigated. Studies that were not relevant to this study were excluded.

### Study selection

The articles' eligibility was determined from each title, abstract, and full text by two reviewers (DP and DS). DP and DS also independently screened the articles for inclusion and extracted data on general information. To solve any disagreements and problems during the study, regular meetings were held by the researchers to discuss issues.

### Data extraction and analysis

The search strategy and inclusion and exclusion criteria were validated and implemented. The initial database was then created from the electronic search. All citations were first filtered by title and abstract, and duplicates omitted. The full texts of eligible papers were then obtained independently for further filtering. After resolving the differences in data extraction or interpretation through consensual discussions based on the inclusion and exclusion criteria mentioned above, the final papers were selected.

The data from the chosen eligible studies were the authors, study period, year of publication, the country where it was conducted, study period, publisher, settings, location characteristics, bioassay methods, the sample of *Anopheles* mosquito, and habitat. The findings were arranged according to the objective and results obtained in related implications of malaria vector control resistance. Throughout the entire selection process, the use of insecticides in the bioassay method, the associated mortality rate of the *Anopheles* mosquito, and its implementation in the specific areas were reported to illustrate the practice's pattern and extent.

All variables for which we extracted data ware Anopheles species, vector habitat, bioassay method, insecticides, mortality rate, insecticide resistance strategies/intervention. The differences in methods could bias the results; to reduce this bias, we selected articles with a similar method. For articles about insecticide resistance, we only looked at articles using bioassay with the world health organization (WHO) standard ^13^. Even though currently the CDC bottle assay is also used for insecticide resistance testing and monitoring, there was no selected articles used CDC bottle assay for testing insecticide resistance and monitoring. The WHO bioassay is carried out with paper impregnated from four main classes of insecticides in common use, with different concentrations according to the WHO test procedure ^14^.

## Results

**Figure 1.**
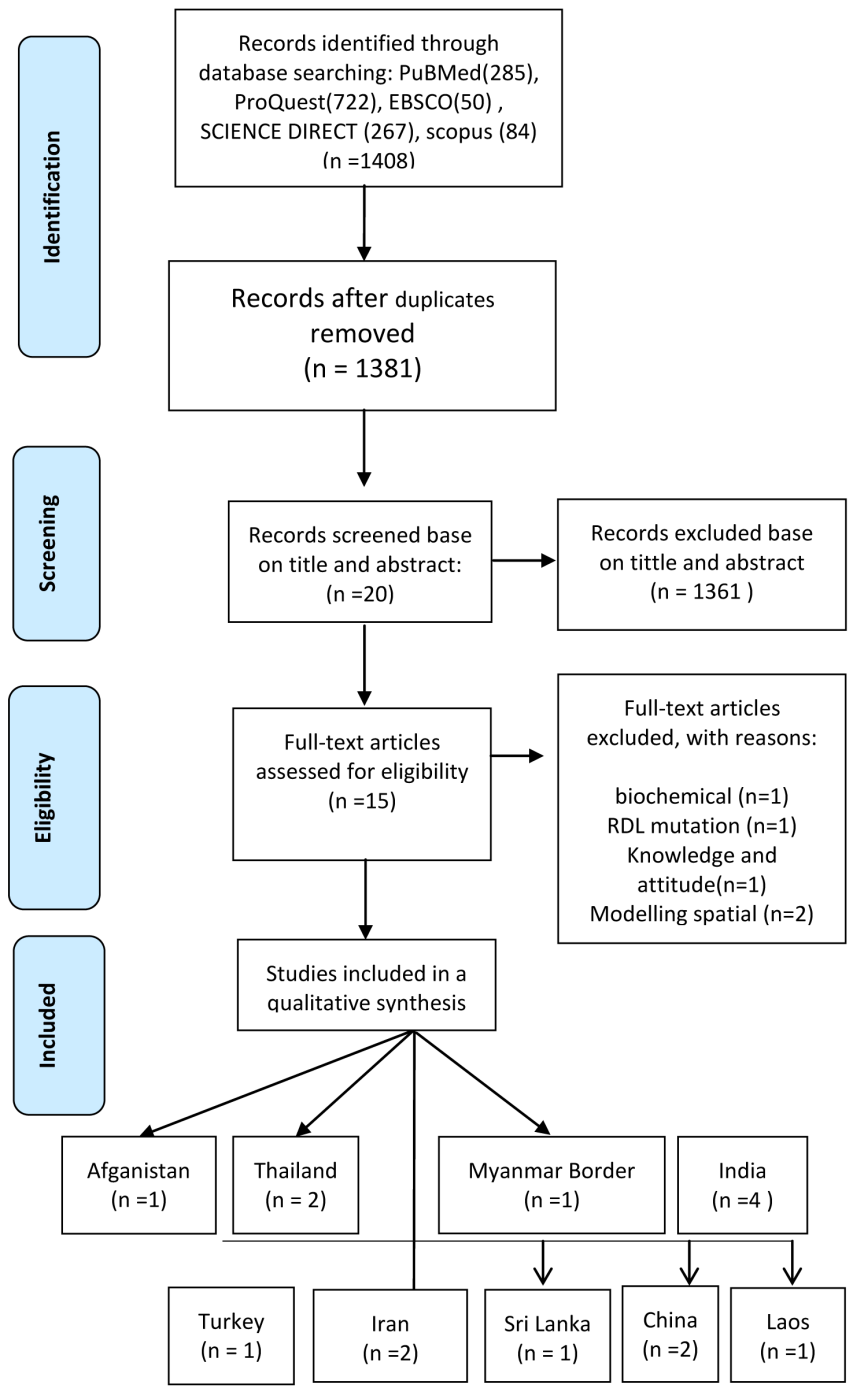
PRISMA flow diagram of systematic review inclusion and exclusion process.

A map showing the countries where insecticide resistance has been reported and the recorded resistance status for each insecticide used is missing shown in Figure 1 ^15^. There were 1,408 articles retrieved during the initial searching (ProQuest=722, Science Direct=267, EBSCO=50, PubMed=285 and Scopus=84). Through screening, 27 articles were excluded because of duplication, 1,361 based on title and abstract incompatibility and 20 due to inconsistency with the inclusion criteria; 15 were chosen to be analyzed.

The 15 eligible articles originated from eight Asian countries published from 2012 to 2019 journals are shown in Table 1.

**Table 1.** Article describtion.

Authors	Title	Year of piblication	Country	Study periods	Publisher	Reference
Ahmad *et al.*	Status of insecticide resistance in high-risk malaria provinces in Afghanistan.	2016	Afghanistan	August to October 2014	Malaria Journal	20
Mishra *et al.*	Insecticide resistance status of *Anopheles culicifacies* in Madhya Pradesh, central India	2012	India	August to September 2009	Journal of Vector- Borne Diseases	8
Dhiman *et al.*	Insecticide resistance and human blood meal preference for *Anopheles annularis* in Asom-Meghalaya border area, northeast India	2014	India	June–August 2011	Journal of Vector- Borne Diseases	21
Sahu *et al.*	Triple insecticide resistance of *Anopheles culicifacies*: A practical impediment for malaria control in Odisha State, India.	2015	India	April to June 2014	Indian Journal of Medical Research	19
Chand *et al.*	Insecticide resistance status of *An. culicifacies* in Gadchiroli (Maharashtra) India	2017	India	August 2016 and February 2017	Pathogens and global health	22
Chareonviriyaphap *et al.*	Review of insecticide resistance and behavioral avoidance of human diseases by vectors in Thailand	2013	Thailand	2000–2010	Parasites & Vectors	23
Chaumeau *et al.*	Insecticide resistance of malaria vectors along the Thailand-Myanmar border	2017	Thailand	August and November 2014, July 2015	Parasites & Vectors	24
Sumarnrote *et al.*	Insecticide resistance status of *Anopheles* mosquitoes in Ubon Ratchathani province, Northeastern Thailand	2017	Thailand	September 2013–September 2015	Malaria Journal	17
Gorouhi *et al.*	Biochemical Basis of Cyfluthrin and DDT Resistance in *Anopheles stephensi* (Diptera: Culicidae) in Malarious Area of Iran	2018	Iran	April–June 2015	Journal of Arthropod-Borne Diseases	25
Vatandoost *et al.*	Indication of pyrethroid resistance in the main malaria vector, *Anopheles stephensi* from Iran	2012	Iran	Spring 2011	Asian Pacific Journal of Tropical Medicine	26
Marcombe *et al*.	Insecticide resistance status of malaria vectors in Lao PDR	2017	Lao	The rainy (June to October) and dry (January to May) seasons of 2014 and 2015	PloS One	27
Qin *et al*.	Insecticide resistance of *Anopheles sinensis* and An. vagus in Hainan Island, a malaria-endemic area in China	2014	China	July–August 2012	Parasites & Vectors	28
Dai *et al.*	Development of insecticide resistance of malaria vector *Anopheles sinensis* in Shandong Province In China	2015	China	2003–2012	Malaria Journal	29
Surendran *et al.*	Variations in susceptibility to common insecticides and resistance mechanisms among morphologically identified species of the malaria vector *Anopheles subpictus* in Sri Lanka	2012	Sri Lanka	July 2008–June 2010	Parasites & Vectors	30
S.İ. Yavaşoglu, *et al.*	Current insecticide resistance status of *Anopheles sacharovi* and *A. superpictus* in former malaria-endemic areas of Turkey	2019	Turkey	April 2014 and September 2015	ActaTropica	31

There were 23 species of *Anopheles* from these studies ( Table 2). The main vectors included *An. stephensi* (Iran), *An. superpictus* (Afghanistan), *An. culicifacies* (India), *An. minimus* and *An. maculatus* (Lao and Thailand), *An. sinensis* (China), *An. subpictus* (Sri Lanka), and *An. sacharovi* (Turkey). From Table 2, the habitat of malaria vector was divided into four habitats: 1) Agriculture: rice fields (paddy fields), 2). Mountains (forest), 3). Aquatic habitat (rivers, ponds, streams, swamps), and 4) Coastal (seaport). In India, *An. cullicifatus* was found only in the forest, but *An. annularis* was found in the forest an irrigation pond. *Anopheles cullicifatus* in Afghanistan was found together with *An. stephensi* and *An. superpictus* at agriculture and aquatics habitat. *Anopheles annularis* was also found in Thailand on Agriculture (paddy fields). In Iran, there was only *An. stephensi* was found in coastal areas and ports. In coastal and inland Sri Lanka was discovered *An. subpictus* and *An. sundaicus*. In Thailand and Lao, many species were found in forests and agriculture (paddy fields) such as *An. annularis*, *An. minimal*, *An. hyrcanus*, *An. barbirostris*, *An. vagus*, *An. maculatatatus*, *An. jamessi*, *An. scanloni*, *An. kochi*, *An. tesselatus*, *An. dirus*, *An. karwari*, *An. nivpes*, *An. vagus*, *An. philipinensis*. As same as in Thailand, in Lao there were also many species of *Anopheles* in the same habitat, except *An. umbrosus* and *An. aconitus* were in Lao and An. jamessi, An. scanloni in Thailand. In China, there were two species in mountains, aquatics habitat, and agriculture; they were *An. sinensis* and *An. vagus.* *Anopheles superpictus*, besides being found in Afghanistan, also in Turkey together with *An. sacharowi* on the farm, waters, and swamps. The differences in the main vector of each country depended on environmental/ecological conditions, living habitat, as well as the feeding and resting behavior of each *Anopheles*.

**Table 2.** Mosquito species and their types of habitat.

Study location	Country	*Anopheles* species	Sample adult female mosquitos (n)	Vector habitat	Reference
Nangarhar, Laghman, Kunar, Ghazni, and Badakhshan	Afghanistan	*An. stephensi*, *An. superpictus*, *An. culicifacies*	2049	Ricefield, river stream, ponds, and water puddle	20
Madhya Pradesh	India	*An. culicifacies*	NA	Forest	8
Asom-Meghalaya border area, northeast India	India	*An. annularis*	200	Forest, ponds irrigation	21
Rayagada, Nowrangpur, Kalahandi, Malkangiri and Koraput	India	*An. culicifacies*	1740	Forest	19
Gadchiroli district	India	*An. culicifacies*	NA	Forest	22
Chiang Mai-Chiang Dao, Mae Hongsom, Phrae	Thailand	*An. minimus* *An. annularis*	NA	Paddy fields and rivulet	23
Thailand-Myanmar Border	Thailand	*An. annularis*, *An. minimus*, *An. hyrcanus*, *An. barbirostris*, *An. vagus*, *An. maculatus*, *An. jamessi*, *An. scanloni*, *An. kochi*, *An. tesselatus*	5896	Agriculture	24
Khong Chiam, Sirindhorn, Buntharik, and Nachaluay	Thailand	*An. hyrcanus*, *An. barbirostris*, *An. maculatus*, *An. nivipes*, *An. philipinensis*, *An. vagus* *An. dirus* *An. karwari*	2088	Forest and ricefield	17
Chabahar Seaport, southeast corner of Iran	Iran	*An. stephensi*	317	Seaport	25
Sistan and Baluchistan	Iran	*An.stephensi*	733	Coastal	26
Phongsaly, Bokeo, LuangPrabang, Vientiane Pro, Borlikhamxay, Khammouane, Savannakhet, Saravane, Sekong, Attapeu.	Lao	*An. minimus*, *An. hyrcanus*, *An. vagus* *An. maculatus* *An. nivipes* *An. philipinesnis* *An. umbrosus* *An. kochi* *An. tesselatus* *An. aconitus*	3977	Forest, village, agriculture	27
Hainan Island	China	*An. sinensis*, *An. vagus*	1468	Mountainous and ricefield	28
Shandong Province	China	*An. sinensis*	4370	Irrigated ricefield, aquatic habitat, and small ponds	29
Batticaloa, Puttalam, Trincomalee and Ampara	Sri Lanka	*An. subpictus* *An. sundaicus*	256	Coastal and inland	30
Southeastern Anatolia and the Mediterranean	Turkey	*An. superpictus*, *An. sacharovi*	1230	Agricultural, ponds, stream and swamps	31

All the female *Anopheles* collected were morphologically identified for their species/complexes using stereomicroscopes and morphological keys ^16^. The mosquitoes were separated by species/complexes for bioassays. The mosquitoes kept alive by giving them a sugar solution ^17^.

*Anopheles* mosquitoes were morphologically identified at the adult stage using the Glick identification key ^18^. The susceptibility tests were carried out following the WHO guidelines for monitoring resistance in malaria vectors. From 15 papers reviewed, the papers impregnated with insecticides of DDT (4%), malathion (5%), bendiocarb (0.1%), propoxur (0,1%), deltamethrin (0.05%) and l-cyhalothrin (0.05%), cyfluthrin 0.15%, permethrin 0.75%, and etofenprox 0.5% were prepared by adopting the WHO standard method ^14^ ( Table 3).

**Table 3.** The WHO bioassay method.

Country	WHO bioassay with insecticides								
	Organochlorine	Organophosphate	Carbamate		Pyrethroid				
	DDT (%)	malathion (%)	Bendiocarb (%)	Propoxur (%)	Permethrin (%)	Deltamethrin (%)	Lambda- cyhalothrin (%)	Cyfluthrin (%)	Etofenprox (%)
Afghanistan	4.0	5.0	0.1	NA	0.75	0.05	NA	NA	NA
India	4.0	5.0	NA	NA	NA	0.05	NA	NA	NA
India	4.0	NA	NA	NA	NA	0.05	NA	NA	NA
India	4.0	5.0	NA	NA	NA	0.05	NA	NA	NA
India	4.0	NA	NA	NA	0.75	0.05	0.05	0.15	NA
Thailand	4.0	NA	NA	NA	NA	NA	NA	NA	NA
Thailand	4.0	NA	NA	NA	0.75	0.05	NA	NA	NA
Thailand	4.0	NA	NA	NA	0.75	0.05	NA	NA	NA
Iran	4.0	NA	NA	NA	0.75	0.05	0.05	0.15	0.5
Iran	4.0	NA	NA	NA	0.75	0.05	0.05	0.15	0.5
Lao	4.0	NA	NA	NA	0.75	0.05	NA	NA	NA
China	4.0	5.0	NA	NA	NA	0.05	NA	NA	NA
China	4.0	5.0	NA	NA	NA	0.05	NA	0.15	NA
Sri Lanka	4.0	5.0	NA	NA	NA	0.05	0.05	NA	NA
Turkey	4.0	5.0	NA	0,1	0,75	0,05	NA	NA	0.5

WHO=world health organization. DDT=dichlorodiphenyltrichloroethane. NA= not available.

The insecticide bioassay was then carried out using a recommended standard WHO kit ^13^. The mortality rate was recorded 24 hours after exposure, while the average death was calculated for each insecticide and according to the WHO criteria ^19^. Bioassy results according to the WHO citeria are susceptible (≥98% mortality), possible resistance (90–97% mortality) or confirmed resistance (<90% mortality ^13^.

The highest mortality rate (MR) ≥ 98% of etofenprox application was on *An. stephensi* in Iran. While permethrin application was on *An. superpictus* (Afghanistan), *An. nivipes* (Thailand), *An. philipinensis* (Lao and Thailand), and *An. tesselatus (*Lao). Then, bendiocarb and malathion were on *An. culicifacies* (India and Afghanistan). Also, deltamethrin was on *An. anularis* (India), *An. barbirostris*, *An. dirus*, *An. karwari* (Thailand), *An. vagus* (Thailand and China), *An. maculatus* (Lao and Thailand), and *An. umbrosus* (Lao). *An minimus* (Thailand Myanmar Border), Meanwhile, permethrin and deltamethrin were on *An. aconitus*, and *An. kochi* (Lao). Lastly, lambda cyalothrin and deltamethrin were on *An. sundaicus* (Sri Lanka), with MR ≤ 97% indicating resistance-possibility based on the WHO classification.

Table 4 shows the level of *anopheles* resistance to organochlorine (dichlorodiphenyltrichloroethane; DDT), organophosphate (malathion), carbamate (bendiocarb and propoxur), and pyrethroid (permethrin, deltamethrin, lambda cyalothrin, cyfluthrin, and etofenprox). Almost all the species of this mosquito studied were possibly resistant to DDT. Furthermore, this similar issue has been reported in *An. stepensi*, *An. superpictus*, *An. culicifacies*, *An. vagus*, *An. sinensi*, *An. subpictus*, and *An. sachrovi* to malathion. Also, it was found in *An. superpictus* and *An. sachrovi* to propoxur as well as in *An. umbrosus* to permethrin. The same was in *An. sinensis*, *An. superpictus*, *An. sundaicus*, *An. minimus*, *An. maculatus* and *An. jamessi* to deltamethrin. Resistance was also reported in *An. stephensi*, *An. culcifacies*, *An. vagus*, and *An. barbirostris* to permethrin and deltamethrin, and in *An. stephensi* to etofenprox. However, direct resistance was found in *An. hyrcanus* to Permethrin and deltamethrin, as well as in *An. culicifacies* to lambda cyalothrin. Also, this was found in *An. stephensi*, *An. sinensis* and *An. culicifacies* to cyfluthrin.

**Table 4.** Anopheles mortality rates in insecticide resistance bioassays.

No	*Anopheles* species	Percentage of mortality (Susceptibility Status)									Reference
		Organochlorine	Organophosphate	Carbamate		Pyrethroid					
		DDT (%)	Malathion (%)	Bendiocarb (%)	Propoxur (%)	Permethrin (%)	Deltamethrin (%)	Lambda- cyhalothrin (%)	Cyfluthrin (%)	Etofenprox (%)	
1	An. stephensi ^*^	31-60 **(R)**	47-97 **(R)**	87-100 (PR)	NA	87-91 (PR)	66-78 **(R)**				20
		45 **(R)**	NA	NA	NA	92.3 (PR)	96(PR)	88.4 (PR)	55 **(R)**	91 (PR)	26
		62 **(R)**	NA	NA	NA	NA	96 (PR)	89 (PR)	82 (PR)	100 (S)	25
2	An. superpictus ^*^	50-86.7 (R-PR)	61.7-88.3 (R-PR)		68.3-91.7 (R-PR)	NA	NA	NA	NA	NA	31
		100 (S)	100 (S)	92 (PR)	NA	100 (S)	85 (PR)	NA	NA	NA	20
3	An. culicifacies ^*^	6.6-26.6 **(R)**	65.4-100 (R-S)	NA	NA	NA	71.6-94.1 (R-PR)	NA	NA	NA	8
		11.4-15.3 **(R)**	60.4-76.2 **(R)**	NA	NA	NA	72.6-84 (R-PR)	NA	NA	NA	19
		37.1 **(R)**	74 **(R)**	NA	NA	91.3 (PR)	83.8 (PR)	59.9 **(R)**	70.2 **(R)**	NA	22
		81 (PR)	95 (PR)	100 (S)		89 (PR)	64 **(R)**				20
4	*An. annularis*	11.9-28.3 **(R)**	NA	NA	NA	NA	97.7-98.1(PR-S)	NA	NA	NA	21
		NA	NA	NA	NA	NA	NA	NA	NA	NA	23
							100 (S)				24
5	An. minimus ^*^	NA	NA	NA	NA	NA	NA	NA	NA	NA	23
		NA	NA	NA	NA	NA	92 (PR)	NA	NA	NA	24
		98-100 (S)	NA	NA	NA	100 (S)	100 (S)	NA	NA	NA	27
6	An. hyrcanus ^*^	57 **(R)**	NA	NA	NA	48 **(R)**	33 **(R)**	NA	NA	NA	24
		72-83 (R-PR)	NA	NA	NA	65-87 (R-PR)	45-85 (R-PR)	NA	NA	NA	17
		90 (PR)	NA	NA	NA	NA	NA	NA	NA	NA	27
7	An. barbirostris ^*^	69 **(R)**	NA	NA	NA	NA	97-100 (PR-S)	NA	NA	NA	17
		74 **(R)**	NA	NA	NA	84 (PR)	72 **(R)**	NA	NA	NA	24
8	An. vagus ^*^	34-61 **(R)**	NA	NA	NA	89-95 (PR)	79-95 (R-PR)	NA	NA	NA	27
		67.1-88.8 (R-PR)	77.3-88.9 (R-PR)	NA	NA	NA	NA	NA	NA	NA	28
		97 (PR)	NA	NA	NA	95 (PR)	75 **(R)**	NA	NA	NA	24
		NA	NA	NA	NA	NA	97.9-100 (S)	NA	NA	NA	28
		NA	NA	NA	NA	NA	100 (S)	NA	NA	NA	17
9	An. maculatus ^*^	86-100 (PR-S)	NA	NA	NA	NA	NA	NA	NA	NA	27
		NA	NA	NA	NA	97 (PR)	85 (PR)	NA	NA	NA	24
		NA	NA	NA	NA	NA	100 (PR)	NA	NA	NA	27
		NA	NA	NA	NA	NA	100 (PR)	NA	NA	NA	17
10	*An. jamessi*	NA	NA	NA	NA	NA	87 (PR)	NA	NA	NA	24
11	*An. nivipes*	0-100 (R-S)	NA	NA	NA	90-100 (PR)	100 (S)	NA	NA	NA	27
		NA	NA	NA	NA	100 (S)	NA	NA	NA	NA	17
12	*An. philippinenses*	33-100 (R-S)	NA	NA	NA	100 (S)	NA	NA	NA	NA	27
		100 (S)	NA	NA	NA	100 (S)	100 (S)	NA	NA	NA	17
13	*An. umbrosus*	63 **(R)**	NA	NA	NA	86 (PR)	100 (S)	NA	NA	NA	27
14	An. sinensis ^*^	30.4 **(R)**	86.6 (PR)	NA	NA	NA	35.8 **(R)**	NA	32.4 **(R)**	NA	29
		72.7-78.4 **(R)**	NA	NA	NA	NA	85.8-91(PR)	NA	NA	NA	28
15	An. subpictus ^*^	16-35 **(R)**	49-69 **(R)**	NA	NA	NA	82-96 (PR)	72-97 (R-PR)	NA	NA	30
16	An. sacharovi ^*^	55-78.3 **(R)**	58.3-90 (R-PR)	NA	68.3-90 (R-PR)	NA	NA	NA	NA	NA	31
17	*An.scanloni*	84 (PR)	NA	NA	NA	NA	NA	NA	NA	NA	24
18	*An.kochi*	82-100 (PR-S)	NA	NA	NA	100 (S)	100 (S)	NA	NA	NA	27
		NA	NA	NA	NA	NA	98 (S)	NA	NA	NA	24
19	*An.tessellatus*	14 **(R)**	NA	NA	NA	100 (S)	NA	NA	NA	NA	27
		NA	NA	NA	NA	NA	98 (S)	NA	NA	NA	24
20	*An.sundaicus*	38-47 **(R)**	93-98 (PR-S)	NA	NA	NA	97-100 (S)	100 (S)	NA	NA	30
21	*An.aconitus*	100 (S)	NA	NA	NA	100 (S)	100 (S)	NA	NA	NA	27
22	*An.dirus*	NA	NA	NA	NA		100 (S)	NA	NA	NA	17
23	*An.karwari*	NA	NA	NA	NA		100 (S)	NA	NA	NA	17

*Main vector DDT=dichlorodiphenyltrichloroethane. NA=not available, S=Susceptible (90-97% mortality suggest), P=Possible Resistance, R=Resistance= < 90%.

Table 5 shows that the insecticide resistance management strategies in several Asian countries are through vector control by environmental, biological, and chemical interventions. The implementation of chemical interventions is through insecticide rotation, monitoring their bioefficacy, mapping, and surveillance of malaria vectors. Malaria eradication relies on effective prevention, technical capability approaches, government and community support, funding sources, accurate data, and adequate implementation.

**Table 5.** Insecticide resistance management strategies based on *Anopheles* habitat.

Country	Study location	habitat	Insecticide resistance strategies	Reference
Afganistan	Nangarhar, Laghman, Kunar, Ghazni, and Badakhshan	Rice field, river stream, ponds, and water puddle	Establishing a management plan for insecticide resistance, and monitoring this situation in all malaria-endemic provinces.	20
India	Madhya Pradesh	Forest	Resistance management strategy by appropriate rotation of different insecticides, including carbamates and incorporating a synergist with synthetic pyrethroids for treating mosquito nets for the control of malaria vectors in these areas. Periodical monitoring of susceptibility/ resistance status of different insecticides.	8, 19, 21, 22
	Asom-Meghalaya border area, northeast India	Forest, ponds irrigation		
	Rayagada, Nowrangpur, Kalahandi, Malkangiri and Koraput	Cattle sheds, human dwelling		
	Gadchiroli district	Forest		
Thailand	Chiang Mai-Chiang Dao, Mae Hongsom, Phrae	Paddy fields and rivulet	Vector prevention strategies and monitoring insecticide resistance. Achieving universal coverage and proper use of LLIN for all people at risk of malaria. Alternative control tools (e.g., insecticide-treated clothes, spatial repellents, or treated hammocks) adapted to the situation of people's activities are more effective in reducing the malaria burden	17, 23, 24
	Thailand-Myanmar Border	Agriculture		
	Khong Chiam, Sirindhorn, Buntharik, and Nachaluay	Forest and rice field		
Iran	Chabahar Seaport, southeast corner of Iran	Seaport	Biological, chemical, and environmental management. Rotation of insecticide. Monitoring and mapping of insecticide resistance in the primary malaria vector for the implementation of any vector control. Evaluation of the mechanisms and implementation of proper insecticide resistance management strategies.	25, 26
	Sistan and Baluchistan	Coastal		
Lao	Phongsaly, Bokeo, LuangPrabang, Vientiane Pro, Borlikhamxay, Khammouane, Savannakhet, Saravane, Sekong, Attapeu.	Forest, village	Routine monitoring of the insecticide resistance levels and mechanisms to ensure effective malaria control. Use of insecticide with different modes of action, rotation, or combination in the same area.	27
China	Hainan Island	Mountainous and ricefield	Cost-effective integrated vector control programs that are beyond synthetic insecticides. The genetic basis of insecticide resistance to implementing more effective vector control strategies. Monitoring the efficacy of common insecticide and exploring the molecular basis of resistance.	28, 29
	Shandong Province	Irrigated ricefield, aquatic habitat, and small ponds		
Sri Lanka	Batticaloa, Puttalam, Trincomalee and Ampara	Coastal and inland	Monitoring genetically different vector populations and their sensitivity to varying insecticides. Developing simple molecular tools and techniques to differentiate morphologically similar *Anopheles* species on the field.	30
Turkey	Southeastern Anatolia and the Mediterranean	Agricultural, ponds, stream, and swamps	Effective management of insecticide resistance and monitoring of the status at a regular interval to prevent delay to its development. Integrated vector control strategies including biological, chemical, and physical strategies implemented in a combination	31

## Discussion

### Study sites

Ecologically, the sites used were mountainous, harbor/seaport, mixed thicket/ lush and dense forests, humid climate, rivers, rice fields, and ponds that provide a suitable environment for vector mosquito breeding. *Anopheles* mosquitos' seasonal activity differs in various regions due to environmental conditions ^24^. Also, those collected were identified for species based on their morphological characteristics ^19, 21^.

### Type of insecticides

Almost all *Anopheles* in this study were reported to be resistant to DDT. Malathion (organophosphate) is still quite effective on *Anopheles sundaicus* 93% in Sri Lanka and *Anopheles superpictus* 100% in Afghanistan. Carbamate are still quite effective for *Anopheles superpictus* 92% in Afghanistan. Pyrethroids were still quite effective with a range of 97–100% in *An. superpictus* (Afghanistan), *An. maculatus* (Lao and Thailand), *An. nivipes* (Lao and Thailand), *An. philipinenses* (Lao and Thailand), *An. minimus*, *An. kochi*, *An. teselatus* and *An. aconitus* (Lao), *An. karwari* and *An. vagus* (Thailand) and *An. annularis* (Thailand-Myanmar border). The application of chemical insecticides is one of the most critical interventions for malaria control, which included organochlorines (DDT, dieldrin, and BHC), organophosphates (pyrimytophos-methyl and malathion), carbamates (propoxur), and pyrethroids (lambda-cyhalothrin and deltamethrin). These chemicals were used in various forms of application, such as indoor residual spraying (IRS) and insecticide-treated mosquito nets (ITNS) for controlling adult mosquitos. In contrast, organophosphates for larviciding were used in malaria-prone areas ^25^. Actually, the pyrethroids were used in various Asia countries for ITNs and long-lasting insecticidal nets (LLINs). They were also considered the most effective because of their advantages, namely low mammalian toxicity, rapid knockdown activity, and high efficacy against a wide range of insect pests, especially mosquitos ^25^.

### Insecticide resistance level frequencies

Resistance to various insecticide, especially to DDT and pyrethtoids, was common problem in different malaria vector species ^32^. The multiple resistance to organochlorine, organophosphate and pyrethroid in this study was reported in *An. stephensi* (Afghanistan) and *An. culicifacies* (India). *An. hyrcanus* and *An. barbirostris* (Thailand-Myanmar border) and *An. sinensis* (China) were multiple resistant to organochlorines and pyrethroids, while *An. subpictus* in Sri Lanka was multiple resistant to organochorine and organophosphate. In Turkey, *An. superpictus* is multiple resistant to organochlorines, organophosphates and carbamates.This multiple resistance was reported in 14 malaria vector in Asia; these included: *An. stephensi*, *An. superpictus*, *An. culinary*, *An. annularis*, *An. minimus*, *An. hyrcanus*, *An. barbirostris*, *An. vagus*, *An. maculatus*, *An. jamessi*, *An. nivipes*, *An. philippinensis*, *An. umbrosus*, and *An. sinensis*. Most of the new reports were towards pyrethroid compounds, the only insecticides used for LLINs ^25^. It represents a growing challenge for malaria control and elimination in the future. Using the same insecticide for multiple successive IRS cycles may not be recommended; it is preferable to use a rotation system with different groups of insecticides, including carbamates ^33^. Rotations should start with the insecticides to which there is the lowest frequency of resistance. In high coverage areas with LLINs, pyrethroids may not be a good option for IRS, as this will add to selection pressure; preferably a rotation system with various types of these groups, including carbamates, should be used ^33^. The rotation should start with an insecticide that has the lowest resistance frequency. In high-coverage areas with LLINs, pyrethroids were good choices for IRS because this added to the selection pressure. Both LLINs and IRS are the most effective insecticides where the local vectors are endophagic and endophilic. But, when the local vectors primary exophagic and exophilic, these interventions still need an essensial level of control ^3^.

Furthermore, an insecticide mixture was a better choice for malaria vector resistance and insecticide resistance management. For example Long-lasting Insecticidal Net (LLIN) incorporating permethrin and a synergist, piperonyl butoxide (PBO), into its fibers in order to counteract metabolic-based pyrethroid resistance of *Anopheles gambiae* *s.s. mosquitoes* ^34^. The efficacy of mixed nets, is because it prevents mosquito bites (function of resistance and physical integrity), and kills mosquitoes (function of chemical content and mosquito susceptibility) ^35^. Resistance has been observed in more than 500 insect species worldwide, among which over 50 *Anopheles* species (Diptera: Culicidae) are responsible for the transmission of malaria parasites to humans ^36^. Since monitoring of the resistance was a critical element for implementing insecticide-based vector control interventions, there was a need for periodic surveillance at least once a year or preferably every six months ^13^. to strengthen the evidence base for the effectiveness of ongoing vector control interventions ^19^. A new insecticide, Sumishield (clothianidin, neonicotinoid) was prequalified for indoor residue spraying by the WHO in 2017, could be an alternative in dealing with multiple insecticide-resistant *Anopheles* ^37^

### Mortality rates of the *Anophele*s populations

The susceptibility and resistance to insecticides are defined based on testing of vector mortality exposure to discriminatory doses: 1) Susceptibility: an observation of more than or equal to the mortality rate of 98% among vectors tested for resitance provides evidence of clear sustainability; 2) Possible resistance: an initial observations of less than 98% of vector mortality in bioassay carried out shows possible resistance. After this observation is made, further testing is needed to confirm resistance. Additional tests must be done to determine whether the vector mortality rates are consistently lower than 98% and to understand resistance levels ^32^. All vectors had resistance to DDT with a value below 80%; however, the use of insecticide began to decline gradually over the last few decades and was removed entirely from malaria control in 2000. This decline was due to the perceived adverse effects on the environment and decreased public acceptance for spraying indoor residues ^23^.

Pyrethroids were the most commonly used insecticides for ITN and IRS, which target indoor transmission and mosquitos that bite in the room ^28^. The mortality rate was <80% for the pyrethroid group in *An. vagus*, *An. culinary*, *An. stephensi*, *An. hyrcanus*, *An. barbirostris*, *An. superpictus*, *An. sacharov*i, and *An. subpictus.* This proved that pyrethroid was less effective. Meanwhile, insecticide resistance was present in malaria vectors in Asia, and the genes spread rapidly throughout the world ^38^. Mosquitoes have two acetylcholinesterase genes (ace-1 and ace-2), but only ace-1 was found to be significantly associated with insecticide resistance ^28^. High insecticide resistance due to insensitive acetylcholinesterase (AChE) has emerged in genes spread of mosquitoes ^39^. In Africa, sublethal doses of pyrethroids for parasite resistance *Plasmodium falciparum* and *An. gambiae s.s.* can interfere with parasite development in mosquitoes, significantly reducing the proportion of infected mosquitoes and the intensity of infection. This mechanism could enable pyrethroid-treated bed nets to prevent malaria transmission despite increased vector resistance ^40^. As resistance genes spread from province to province and country to country, it is of course meaningful and very useful to observe whether and how much this spread is accompanied by an increase in routine reports of malaria incidence as recorded in local health facilities ^37^

### Interventions

Several countries in Asia are implementing an insecticide resistance management (IRM) strategy against malaria vectors following the Global Plan for IRM; this will be more effective with the support of national health system policies and cross-sectoral coordination to achieve malaria-free targets 2030. The use of insecticides to reduce vector populations has become the main strategy for malaria control. Presently, 12 of these insecticides belonging to four chemical classes are recommended by the WHO Pesticide Evaluation Scheme (WHOPES) for IRS ^41^. The nine insecticides used in Asia which recommended by WHO are Bendiocarb, Propoxur, DDT, Malathion, α-Cypermetrhrin, Cyfluthrin, Deltamethrin, Etofenprox, and Lambda-Cyhalothrin. Current strategies for controlling malaria vectors mainly include IRS with synthetic DDT/pyrethroids and durable LLINs ^14^. WHO recommends that these insecticides' susceptibility status needs to be monitored annually ^13^. However, the last two decades have seen the use of insecticides everywhere, especially pyrethroids, causing widespread resistance and compromising the effectiveness of vector control ^42^. Besides, when this situation is detected, the intensity, biochemical and molecular mechanisms should also be investigated. The accurate information about the underlying resistance mechanism and its intensity or frequency in the malaria vector turns to update the vector control program and ensure the timely management of insecticide resistance ^32^. Therefore, biochemical and molecular tests are recommended to understand the mechanism of pyrethroid resistance, and there have been several reports about this situation in malaria vectors. However, several control strategies are used to overcome resistance, such as rotation, mixture, using biological control, and integrated vector management ^26^.

### Limitations

This study had limitations, such as the dissimilar variables investigated, which produced an incomplete analysis. Only 15 articles from eight countries that correlated with the inclusion criteria from the selected ten years of studies. The data on each country's mortality rate presented only the smallest value, therefore, making it difficult to explore the whole data that needed to make the discussion complete. In some countries, the bioassay test did not use carbamate, even though it was effective for controlling certain types of *Anopheles*.

## Conclusion

This review found organochlorine (DDT), organophosphate (malathion), and pyrethroids resistance in several *Anopheles* species with a less than 80% mortality rate. The reports of pyrethroid resistance were quite challenging because it is considered effective in the malaria vector control. Several countries in Asia are implementing an insecticide resistance management (IRM) strategy against malaria vectors following the Global Plan for IRM. Intervention and implementation with optimal resource support are carried out in several Asian countries, including the management plans in selecting insecticides, using a rotation system during interventions in the field, regular monitoring, and integrating vector control based on physics, chemistry, and biology. Several strategies are needed, including management plans in selecting insecticides, using a rotation system during the field interventions, regular monitoring, and integrating vector control strategies based on physical, chemical, and biological methods. All these need to be supported by cross-sector policies and cooperation to achieve the 2030 malaria-free target.

## Data availability

### Underlying data

All data underlying the results are available as part of the article and no additional source data are required.

### Extended data

Figshare: PRISMA 2009 checklist for an article entitled Current status of insecticide resistance in malaria vectors in the Asian countries: systematic review. https://doi.org/10.6084/m9.figshare.13586078 ^15^.

This project contains the following extended data:

- PRISMA flow diagram of systematic review inclusion and exclusion process

### Reporting guidelines

Figshare: PRISMA checklist for ‘Current status of insecticide resistance in malaria vectors in the Asian countries: systematic review’. https://doi.org/10.6084/m9.figshare.13582517 ^12^.

Data are available under the terms of the Creative Commons Attribution 4.0 International license (CC-BY 4.0).
