# Supplementary material for: Current status of insecticide resistance in malaria vectors in the Asian countries: a systematic review
Source: F1000Res. 2022 Jan 4;10:200. Originally published 2021 Mar 10. [Version 2] doi: 10.12688/f1000research.46883.2 (PMC8802149; doi:10.12688/f1000research.46883.2)
Supplement: Peer review history

# Peer review: Reviewer response for version 2

**DOI:** 10.5256/f1000research.78974.r118553

**Published:** 2022-01-28

**Authors:** Manaswini Dehuri

**Affiliations:** 1 Department of Veterinary Parasitology, College of Veterinary Science and Animal Husbandry, orissa university of agriculture & technology, Bhubaneswar, India

The scope of the article is good and it is a relevant topic. There are grammatical mistakes and many sentences needs to be rewritten in the article e.g.:

- The 2nd line of introduction needs to be rewritten. In the 2nd paragraph of introduction "mainly" has been misspelt.
- In methods the 6th paragraph may be rewritten for ease of understanding. Last paragraph of methods 'were' is misspelt.
- In results, in the 2nd line" missing" should be deleted.  2nd paragraph of results "From Table 2" needs to be deleted. 5th line, forest "an" to be replaced with 'and'. 3rd paragraph, last line should be deleted. 4th paragraph, 2nd line needs to be re written.

Please check the manuscript thoroughly as there are many spelling and grammatical mistakes.

 Statistical analysis (test of significance) ought to be done for Table 3 & 4.

Are the rationale for, and objectives of, the Systematic Review clearly stated?

Yes

Is the statistical analysis and its interpretation appropriate?

Partly

Are sufficient details of the methods and analysis provided to allow replication by others?

Partly

Are the conclusions drawn adequately supported by the results presented in the review?

Yes

Reviewer Expertise:

Veterinary Entomology, Acarology and Protozoology

I confirm that I have read this submission and believe that I have an appropriate level of expertise to confirm that it is of an acceptable scientific standard, however I have significant reservations, as outlined above.

# Peer review: Reviewer response for version 2

**DOI:** 10.5256/f1000research.78974.r118541

**Published:** 2022-01-26

**Authors:** Josiane Etang

**Affiliations:** 1 Organization for Coordination of the Fight Against Endemic Diseases in Central Africa (OCEAC), Yaoundé, Cameroon; 2 Faculty of Medicine and Pharmaceutical Sciences, University of Douala, Douala, Cameroon

Most of the comments made on the previous version of the manuscript have been addressed. However, there is a need for further improvement of the English language through the text.

 Thank you for your consideration.

Are the rationale for, and objectives of, the Systematic Review clearly stated?

Yes

Is the statistical analysis and its interpretation appropriate?

Not applicable

Are sufficient details of the methods and analysis provided to allow replication by others?

Yes

Are the conclusions drawn adequately supported by the results presented in the review?

Partly

Reviewer Expertise:

Medical entomology

I confirm that I have read this submission and believe that I have an appropriate level of expertise to confirm that it is of an acceptable scientific standard.

# Peer review: Reviewer response for version 1

**DOI:** 10.5256/f1000research.49960.r93580

**Published:** 2021-10-01

**Authors:** Charles M. Mbogo

**Affiliations:** 1 Kenya Medical Research Institute-Wellcome Trust Research Program, Nairobi, Kenya

The authors are reporting on insecticide resistance data compiled through systematic review from Asian countries. The purpose of this systematic review was to understand the current status of insecticide resistance among malaria vectors and the implementation of the interventions.

**Introduction/Background:**

 The background information is sound, and the research question is easily identifiable.  However, a few minor comments:

- Paragraph 2 --- “The prevention of malaria mainly depends on one class of insecticides, namely pyrethroids; however, the increase in resistance to it reduces this treatment’s efficacy”. This sentence is not clear because malaria control may be done using the 4 available classes of insecticides and not the pyrethroids unless one is talking about LLINs.
- Paragraph 3 –states that “Vector control is an essential aspect of a program organized to manage the disease transmitted by this type of **organism**”. Be specific and state what kind of organism is this.

**Methods:**

 The methodologies applied are appropriate and adequately described. The searching process used was also appropriate. However, the authors indicated that they selected the 15 papers based on several criteria of which one included WHO bioassay protocol on the four major insecticide classes and did not consider any other bioassay protocols such as CDC bottle assay.

 Currently, insecticide resistance is measured using WHO bioassay protocol and CDC bottle assay. It is important to check if any of the selected publications had any analysis of resistance including CDC bottle assay. If this is correct, then consider it as it could increase the amount of resistance data.

**Results:**

 The results are coherently described with tables and figures. However, Tables 2, 3, 4, & 5 should have clear and legible descriptions.

- Paragraph 2 …” From Table 2, the malaria vector was divided into three habitats …”. This sentence needs to be rephrased to make it clear. How can mosquito vectors be divided into three habitats or what do you mean? The sentence that follows this in the same paragraph should also be rephrased for clarity.
- Paragraph 3 …. “The entire female Anopheles collected was morphologically identified for their species or complexes using stereomicroscopes and morphological keys” The whole of this paragraph and the next one should be combined and written clearly to convey the necessary message.
- “From 15 papers reviewed, the impregnated with insecticides of DDT (4%)…” Insert the word “papers” before impregnated.
- Paragraph 8 … “Table 5 shows…” Consider revising this paragraph to make it clear.

**Discussion and Conclusion**

 The discussion highlights the different aspects of the findings on the status of insecticide resistance in different Asian countries. These are partly discussed in relation to the current literature and the status of insecticide resistance in the region. However, more discussions are required on the outcomes on the types, levels, and intensity of insecticide resistance and how multiple insecticide resistance was reported in the diversity of malaria vectors present in the region. Below are a few minor issues that need to be addressed:

- Page 12, Paragraph 1: ”This became a challenge for malaria control and elimination, therefore, using the same insecticide for multiple successive IRS cycles is not recommended”. Revise this sentence and the entire paragraph to make it clear.
- Page 12, paragraph 2: “…applications against resistible mosquitoes”. Which are these mosquitoes?
- Page 12, paragraph 4: “This proved that pyrethroid was less effective. Meanwhile, insecticide resistance was present in malaria vectors in Asia, and the genes spread rapidly throughout the world”. This statement is misleading ..what evidence do you have that resistance genes were spreading quickly throughout the world?
- Page 12, paragraph 5: It is important to state some of the 12 interventions mentioned here and their challenges and opportunities they have.

Conclusion:  The conclusion is not fully drawn from the analysis of this systematic review?

Are the rationale for, and objectives of, the Systematic Review clearly stated?

Yes

Is the statistical analysis and its interpretation appropriate?

Not applicable

Are sufficient details of the methods and analysis provided to allow replication by others?

Yes

Are the conclusions drawn adequately supported by the results presented in the review?

Partly

Reviewer Expertise:

Medical Entomology

I confirm that I have read this submission and believe that I have an appropriate level of expertise to confirm that it is of an acceptable scientific standard, however I have significant reservations, as outlined above.

# Peer review: Reviewer response for version 1

**DOI:** 10.5256/f1000research.49960.r87116

**Published:** 2021-06-30

**Authors:** Josiane Etang

**Affiliations:** 1 Organization for Coordination of the Fight Against Endemic Diseases in Central Africa (OCEAC), Yaoundé, Cameroon; 2 Faculty of Medicine and Pharmaceutical Sciences, University of Douala, Douala, Cameroon

The emergence and spread of *Anopheles* resistance to insecticides is a growing issue, that jeopardizes the effectiveness of currently available core malaria vector control tools, i.e. Long Lasting Insecticidal nets (LLINs) and Indoor Residual Spraying (IRS).

 Based on data extracted from 15 original articles selected from four science databases, the authors of this paper came out with a baseline review on malaria vector resistance status in Asia. This resistance concerns 23 anopheline species collected in 8 countries between 2012 and 2019, to 10 insecticides belonging to the four chemical classes mostly used for vector control. Additional information on the ecology and habitat of malaria vectors is also provided. Yet, the authors suggested some resistance management strategies, including the selection of insecticides to be used for vector control, the rotation of the interventions, and integrating vector control methods.

 However, as a limitation, the low number and the content of the articles that were eligible for this study (15 papers) made it difficult to thoroughly investigate the geographic and structural distribution of insecticide resistance in target countries, as well as the underlying resistance mechanisms. It appears that the drawn conclusions are partly supported by the results of the review. Furthermore, several issues should be addressed to improve the manuscript:

**1. Abstract**

- Line one: "remained" may be replaced by "led to"; and "due to" replaced by "which is".
- Line two: The study aims to "review" the insecticide resistance status which was previously determined.
- Conclusion: I would suggest replacing "complicated" with "challenging".

**2. Introduction**

- Paragraph 1: please update the situation of malaria, according to the world malaria report 2020.
- Paragraph 2: please add "partly" before "reversible", because there are many other factors that may hinder the reduction of malaria burden apart from insecticide resistance.
- Paragraph 3: It is not clear which type of organism the authors are talking about in the first sentence of this paragraph. At the end of the paragraph, please change the position of "regular" and place it before "monitoring"; i.e. "regular monitoring".

**3. Methods**

- In the last paragraph, it is said that only papers dealing with the WHO bioassay protocol were considered. Currently, the CDC bottle assay is also used for insecticide resistance testing and monitoring. Adding a separate analysis of resistance data from the CDC bottle assay, if any, could probably increase the amount of resistance data. 

**4. Results**

- Paragraph 1: A map showing the countries where insecticide resistance has been reported and the recorded resistance status for each insecticide used is missing.
- Paragraph 2: Please delete "In several countries" since each species is associated with only one or two countries and not all the countries. Also, it is not clear how a malaria vector can be divided into three habitats; and also which malaria vector the authors are talking about?
- Paragraph 3: Please consider revising the first sentence of this paragraph, e.g. All the female *Anophele*s collected were morphologically identified for their species/complexes...". The last sentence about mosquito processing is also confusing; the authors may consider revising it.
- Paragraph 4: Please include "papers" before "impregnated" and replace "was" with "were".
- Paragraph 5: Please delete "summarized in three resistance classes", this is confusing. The authors may just state that "bioassay results were analyzed according to WHO criteria. Also, the reference N°12 is not specific to WHO bioassay protocol, same as reference n°18; please consider revising the numbering of the references. The reference n°13 is appropriate for this paragraph.
- Regarding the interpretation of the results, the authors should check, which of the WHO protocols they used. In the running WHO protocol which was deriving the revision made in 2018, the criteria for interpretation of bioassay results are different from the criteria presented in this paragraph. In the revised protocol, the resistance threshold is 90%, not 80%.
- Paragraph 6: A mortality rate <97% is wide and also includes confirmed resistance, please specify the lowest mortality rates indicating possible resistance. 
- Paragraph 8: The first sentence may be as follows: "Table 5 shows that the insecticide resistance management strategies in several Asian countries are through vector control by environmental, biological, and chemical interventions. The implementation of chemical interventions is through insecticide rotation, monitoring their bioefficacy, mapping, and surveillance of malaria vectors. Malaria eradication relies on..."

**5: Discussion**

- Here are suggestions for subtitles: Types of insecticides, Insecticide resistance frequencies, Mortality rates of Anopheles populations, Interventions.
- Paragraph 2: Types of insecticides - In the second sentence of the paragraph, please replace "And" with "These chemicals". Also, replace "Currently" with " Actually".
- Paragraph 3: Insecticide resistance frequencies - No need to refer to tables in the discussion.
- The authors may combine the first and the second sentences of this paragraph; e.g. " Resistance to various insecticides, especially to DDT and pyrethroids, was common...vector species. Multiple insecticide resistance was reported in 14 malaria vector species in Asia; these include *A. stephensi*... *A. sinensis*." For the last sentence of this paragraph and the first sentence of the next paragraph, the authors should refer to WHO guidelines for using both LLINs and IRS in specific conditions. Please consider replacing "resistible mosquitoes" with "resistant mosquitoes".
- Paragraph 6: Mortality rates of the *Anopheles* - What is the mortality test? Is it the susceptibility test? The author should consider revising the first sentence of this paragraph. It is not clear how a test can be in a range of 98-100%, 80-97%... etc.
- Paragraph 8: In the second sentence of this paragraph, the authors may replace "interventions belonging to..." with "insecticides belonging to..." and delete "durable" from the next sentence.

The following sentences should also be revised as follows:

- " The accurate information about...tuns...of insecticide resistance".
- "Therefore, biochemical...understand the mechanisms...in malaria vectors".
-  "However, several control strategies...used to overcome resistance...vector management".

**6. Conclusion**

 The following sentences should also be revised as follows:

- The reports of pyrethroid resistance were quite challenging, because..."
- "integrating vector control strategies based on physical, chemical and biological methods".

**7. Tables**

- Table 1. The title of this table may be "Article description" rather than "charactéristics".
- Column 3 may be title "year of publication" rather than "publish".
- Table 2: The title of this table may be "Mosquito species and their types of habitat" rather than "sample and location characteristics".
- Table 4. The title of this table may be " *Anopheles* mortality rates in insecticide resistance bioassays" rather than "Mortality rate of insecticide resistance bioassay in *Anopheles*".
- Table 5: The title of this table may be as follows " Insecticide resistance management strategies".

Are the rationale for, and objectives of, the Systematic Review clearly stated?

Yes

Is the statistical analysis and its interpretation appropriate?

Not applicable

Are sufficient details of the methods and analysis provided to allow replication by others?

Yes

Are the conclusions drawn adequately supported by the results presented in the review?

Partly

Reviewer Expertise:

Medical entomology

I confirm that I have read this submission and believe that I have an appropriate level of expertise to confirm that it is of an acceptable scientific standard, however I have significant reservations, as outlined above.
